# Heme Drives Oxidative Stress-Associated Cell Death in Human Neutrophils Infected with *Leishmania infantum*

**DOI:** 10.3389/fimmu.2017.01620

**Published:** 2017-11-23

**Authors:** Graziele Quintela-Carvalho, Nívea F. Luz, Fabiana S. Celes, Dalila L. Zanette, Daniela Andrade, Diego Menezes, Natália M. Tavares, Claudia I. Brodskyn, Deboraci B. Prates, Marilda S. Gonçalves, Camila I. de Oliveira, Roque P. Almeida, Marcelo T. Bozza, Bruno B. Andrade, Valeria M. Borges

**Affiliations:** ^1^Instituto Gonçalo Moniz (IGM), Fundação Oswaldo Cruz (FIOCRUZ), Salvador, Brazil; ^2^Faculdade de Medicina, Universidade Federal da Bahia (UFBA), Salvador, Brazil; ^3^Instituto Federal de Educação, Ciência e Tecnologia Baiano (IFBaiano), Santa Inês, Brazil; ^4^Instituto de Tecnologia e Pesquisa (ITP), Aracaju, Brazil; ^5^Departamento de Biomorfologia, Instituto de Ciências da Saúde, Universidade Federal da Bahia (UFBA), Salvador, Brazil; ^6^Departamento de Medicina, Hospital Universitário, Universidade Federal de Sergipe (UFS), Aracaju, Brazil; ^7^Departamento de Imunologia, Universidade Federal do Rio de Janeiro (UFRJ), Rio de Janeiro, Brazil; ^8^Multinational Organization Network Sponsoring Translational and Epidemiological Research (MONSTER) Initiative, Salvador, Brazil; ^9^Universidade Salvador (UNIFACS), Laureate Universities, Salvador, Brazil; ^10^Escola Bahiana de Medicina e Saúde Pública, Salvador, Brazil

**Keywords:** *Leishmania infantum*, heme, iron, neutrophils, cell death, heme oxygenase 1

## Abstract

Free heme is an inflammatory molecule capable of inducing migration and activation of neutrophils. Here, we examine the heme-driven oxidative stress-associated cell death mechanisms in human neutrophils infected with *Leishmania infantum*, an etiologic agent of visceral leishmaniasis (VL). We first performed exploratory analyses in a population of well characterized treatment-naïve VL patients as well as uninfected controls, who were part of previously reported studies. We noted a positive correlation between serum concentrations of heme with heme oxygenase-1 (HO-1) and lactate deydrogenase, as well as, a negative correlation between heme values and peripheral blood neutrophils counts. Moreover, *in vitro* infection with *L*. *infantum* in the presence of heme enhanced parasite burden in neutrophils, while increasing the production of reactive oxygen species and release of neutrophilic enzymes. Additional experiments demonstrated that treatment of infected neutrophils with ferrous iron (Fe^+2^), a key component of the heme molecule, resulted in increased parasite survival without affecting neutrophil activation status. Furthermore, stimulation of infected neutrophils with heme triggered substantial increases in HO-1 mRNA expression as well as in superoxide dismutase-1 enzymatic activity. Heme, but not Fe^+2^, induced oxidative stress-associated cell death. These findings indicate that heme promotes intracellular *L*. *infantum* survival via activation of neutrophil function and oxidative stress. This study opens new perspectives for the understanding of immunopathogenic mechanisms involving neutrophils in VL.

## Introduction

Leishmaniasis is an emerging tropical neglected disease and causes an average of 2 million new cases per year (1). The disease is caused by parasites of the genus *Leishmania* and can be associated with different clinical manifestations, such as visceral leishmaniasis (VL), which is lethal if not treated. Hematological manifestations are common in VL, mainly anemia and neutropenia. Anemia is associated with a persistent inflammatory condition, increased peripheral destruction of erythrocytes in the spleen, sometimes predisposing to bleeding (2, 3). These hematological disorders can lead to the degradation of hemoglobin and consequent release of heme.

Heme is a molecule composed of a central iron atom bonded to four porphyrin groups (iron protoporphyrin IX), and it is of vital importance to many biological processes associated with proteins involved in redox activity, such as hemoglobin, cytochrome c, catalases, and peroxidases. Under pathophysiological conditions, heme can be released in excessive amounts by heme proteins as well as during cell death (4). In this context, free heme, being quite hydrophobic, merges in cell membranes and increases cell susceptibility to oxidative stress and generation of reactive oxygen species (ROS) (5). In addition, free heme is also able to induce recruitment, activation, and affect death of leukocytes, especially neutrophils (6–9). Antioxidant mechanisms as well as action of scavenger proteins such as hemopexin and albumin help to prevent or minimize the inflammatory effects of heme (10). A stress-responsive enzyme, heme oxygenase-1 (HO-1), catabolizes heme, releasing biliverdin, carbon monoxide, and iron (5). HO-1 has been implicated in the pathogenesis of several infectious diseases, including leishmaniasis. During *Leishmania* infection, the activation of HO-1 results in heme degradation, avoiding the formation of NADPH-oxidase complex (an enzyme containing grouping heme) and ROS production in the parasitophorous vacuole (11). We have previously demonstrated that induction of HO-1 favors *Leishmania infantum* replication and infection in human and murine macrophages by reduction of its leishmanicidal mechanisms, as nitric oxide and ROS (12). We have also described increased serum levels of HO-1 and total heme in VL patients compared to healthy endemic controls (12, 13).

Neutrophils are essential cells from the innate immune system, are rapidly recruited to inflammatory sites and have been described to play a critical role in *Leishmania* infection (14, 15). Recruitment and stimulation of neutrophils in response to inflammatory stimulus lead to differential expression of activation surface markers and increased degranulation of several enzymes, cytokines, and chemokines, while also induces production of free radicals and antioxidants enzymes, such as superoxide dismutases (SOD) (16). The release of granule proteases is important for neutrophil migration and is partially responsible for their microbicidal activity, as well metalloproteinase 9 (MMP-9), neutrophil elastase (NE), and myeloperoxidase (MPO) (17, 18). Our group has previously demonstrated that neutrophils participate on *Leishmania* killing through the activation and release of TNF-α, NE, and MPO (19–23). Whether these activities are directly associated with differential cell death pathways in *Leishmania*-infected neutrophils are not fully understood.

In the present study, we evaluated the effect of heme on human neutrophils during infection with *L. infantum*. Our results revealed in patients with active VL a positive correlation between serum concentrations of heme and HO-1 or lactate dehydrogenase (LDH, an inflammation-derived tissue damage marker), as well as, a negative correlation between heme values and peripheral blood neutrophils counts. We also observed that *in vitro* infection with *L*. *infantum* in presence of heme enhances neutrophil activation and promotes parasite survival. These findings indicate that heme promotes intracellular *L. infantum* survival via activation of neutrophil function and oxidative stress, in addition to induction of cell death. Increased neutrophil cell death and oxidative consequences could be the basis for the occurrence of neutropenia, which hallmarks VL.

## Results

### Circulating Levels of Heme Are Positively Correlated with Serum HO-1 and LDH Values and Associated with Neutropenia in Human VL

**Figure 1:**
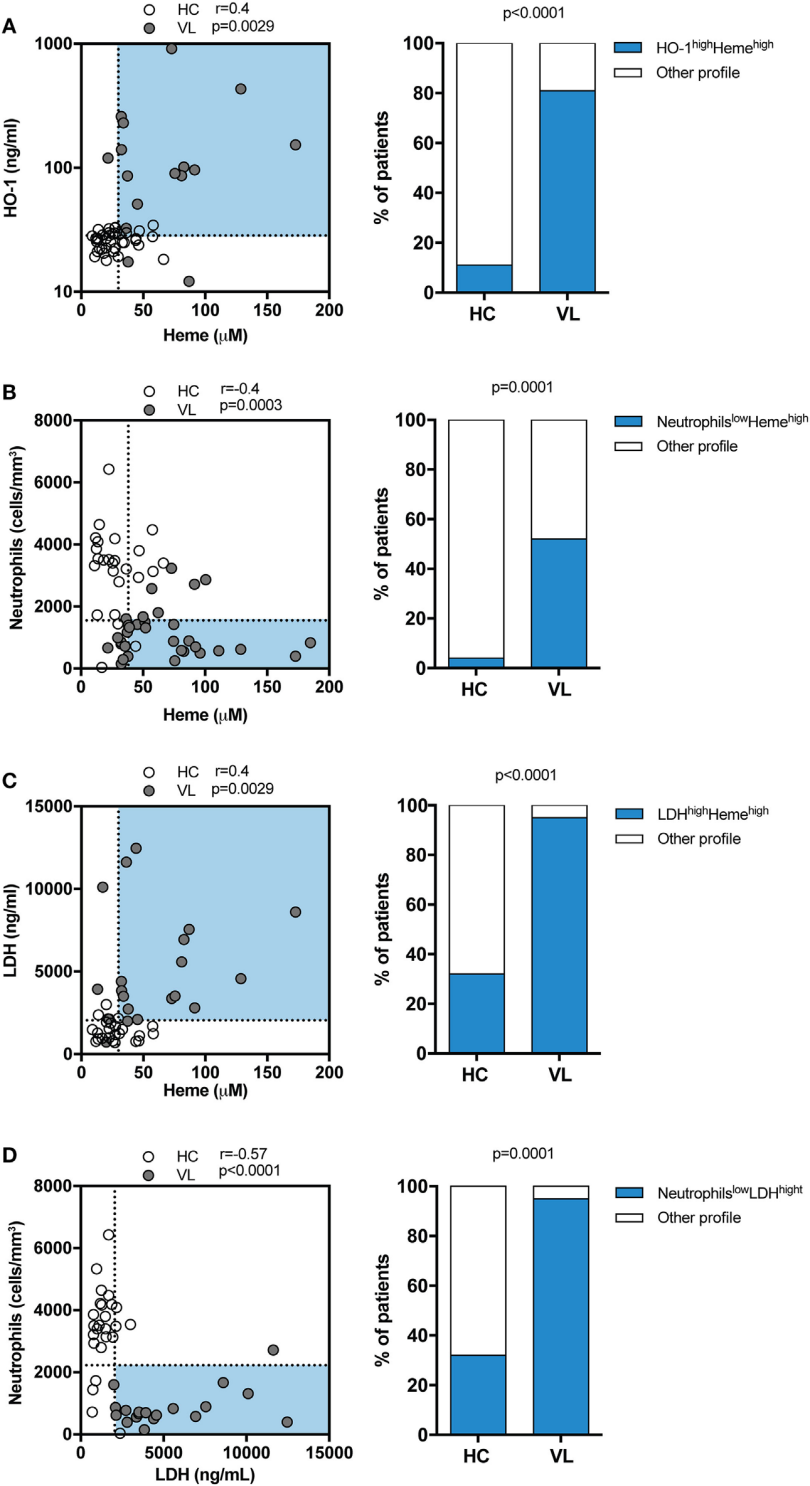
Circulating levels of total heme positively correlate with heme oxygenase-1 (HO-1) and lactate dehydrogenase (LDH) concentrations and associate with neutropenia in patients with visceral leishmaniasis (VL). Serum levels of total heme were tested for correlation with HO-1 **(A)**, peripheral neutrophil counts **(B)** and LDH levels **(C)** using the Spearman test. The dotted lines represent median values of the parameters listed in the *X* or *Y*-axis, respectively. Frequencies of healthy endemic controls (HC) individuals and those with VL who simultaneously displayed high levels of total heme and HO-1 **(A)** or high levels of total heme and low neutrophil counts **(B)** or high levels of total heme and and high levels of LDH **(C)** were compared using the Fisher’s exact test. Plasma levels of LDH were correlated to the peripheral neutrophil counts **(D)** using the Spearman test. Frequencies of healthy endemic controls (HC) individuals and those with VL who simultaneously displayed high levels of LDH and low neutrophil counts **(D)** were compared using the Fisher’s exact test. In the left graphs, each dot represents a single individual.

Both HO-1 and heme have been previously described as important biomarkers associated to VL (12, 13). Serum LDH is usually elevated in hemolytic diseases, reflecting the degree of cell/tissue damage (24). In the present study, we re-examined the data from a patient cohort previously published by our group (12, 13) to assess correlations between circulating levels of total heme with LDH, HO-1, or peripheral blood neutrophils counts. Serum samples were obtained from patients with VL and from sex-matched endemic controls, all from a highly endemic area in the northeast of Brazil. In this study population, heme levels were shown to be positively correlated with HO-1 (*r* = 0.4; *p* = 0.0029; Figure 1A) and about 81% of VL patients exhibited simultaneously high levels of total heme and of HO-1, a condition observed in only 11% of healthy individuals (*r* = 0.04; *p* = 0.0029; Figure 1A). In addition, there was a negative correlation between levels of heme and peripheral blood neutrophil counts (*r* = –0.4; *p* = 0.0003; Figure 1B); with 52% of VL patients exhibiting simultaneously high levels of heme and low neutrophil counts, which was observed in only 4% of healthy individuals. Heme levels were shown to be positively correlated with LDH values (*r* = 0.4; *p* = 0.0029; Figure 1C) and approximately 90% of VL patients exhibited simultaneously high levels of LDH and of heme, a condition observed in only 30% of healthy individuals. Furthermore, we observed a negative correlation between levels of LDH and of peripheral blood neutrophil counts (*r* = –0.57; *p* < 0.0001; Figure 1D). Up to 92% of VL patients exhibited simultaneously high levels of LDH and low neutrophil counts, which was observed in a minority of healthy individuals (30%). These observations argued that heme levels in serum may be directly linked to neutrophil death in the peripheral blood.

### Heme Triggers Cell Death in Neutrophils during *L. infantum* Infection

**Figure 2:**
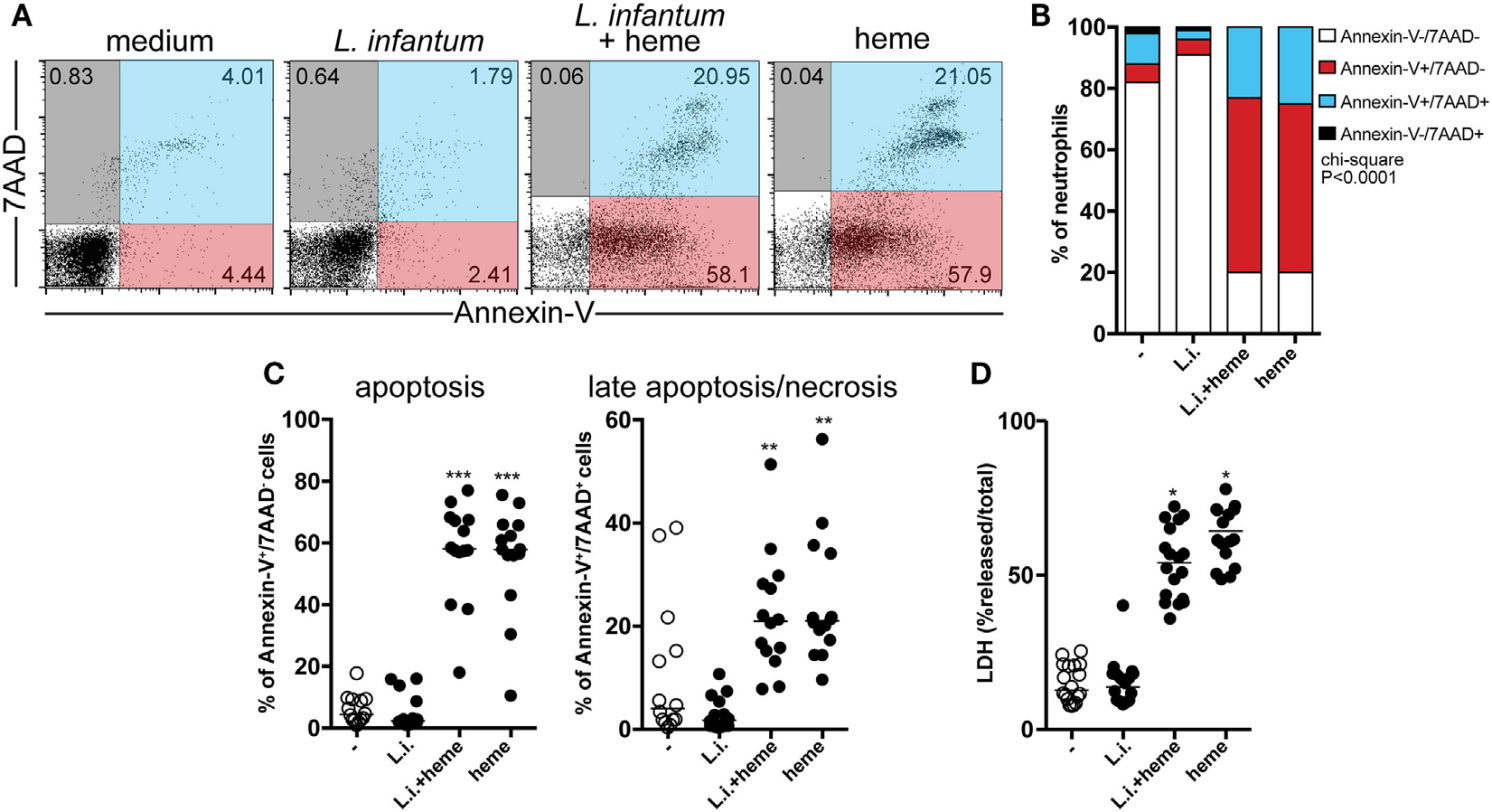
Heme triggers cell death in neutrophils during *L. infantum* infection. Human neutrophils were infected with *L. infantum* in the presence or absence of heme (30 µM) for 3 h. Apoptosis and late apoptosis/necrosis were assessed by staining for both Annexin V and 7-AAD using flow cytometry. Gated neutrophils were analyzed following staining with 7-aminoactinomycin D (7AAD) and Annexin V, the percentage of the cells is given in each quadrant **(A)**. Frequencies of Annexin V^−^/7AAD^−^, Annexin V^+^/7AAD^−^, Annexin V^+^/7AAD^+^, and Annexin V^−^/7AAD^+^ **(B)**. Percentage of apoptotic (Annexin V^+^/7AAD^−^) or late apoptotic/necrotic cells (Annexin V^+^/7AAD^+^). Circles represent individual values **(C)**. **(D)** Total lactate dehydrogenase (LDH) release was determined by lysing the cells with 1% Triton X-100. Each point on the graph represents a donor, and the bars represent the median vales of the percent (%) total LDH release. Asterisk indicates significant differences assessed using non-parametric Kruskal–Wallis test with Dunn’s post-test (**p < 0.01; ***p < 0.001).

Given that we found that heme is related to reduced neutrophil count in patients with VL, we next focused on investigating whether heme is involved in neutrophil death in the context of *L. infantum* infection using an *in vitro* model employing flow cytometry. Using this approach, cells with the staining profile of Annexin-V^+^/7AAD^−^ are considered apoptotic whereas those which are Annexin-V^+^/7AAD^+^ are considered in late apoptosis/necrosis. Human neutrophils were experimentally infected with *L*. *infantum* and incubated in the absence or presence of heme for 3 h. We have used 30 μM of heme in the experiments because this was the average level detected in serum from patients with VL (13), as similar to what has been described in malaria (25, 26) and sickle cell disease (27). In addition, doses close to 30 µM have been commonly used in *in vitro* experiments published from other groups (8, 9, 28). We observed that *L. infantum* infection was not able to significantly induce neutrophil apoptosis, neither necrosis (Figures 2A,C), however in the presence of heme, a higher frequency of the neutrophils became Annexin-V^+^/7AAD^+^ (e.g., in late apoptosis/necrosis) (Figures 2B,C). Noteworthy, our experiments show important donor variability in heme-induced cell death, ranging from 20% (min value) to 80% (max value), and the average% of early apoptosis (Annexin-V^+^/7AAD^−^ cells) was 60%, while the median frequency late apoptotic/necrotic cells (Annexin-V^+^/7AAD^+^ cells) was around 20% (Figure 2C). Following *L. infantum* infection in the presence of heme, there was significant increase in LDH levels released in cell supernatants, indicating augmented cell death (Figure 2D). These findings indicate that heme is tightly associated with increased cell death in neutrophils, but not necessarily only in those infected with *L. infantum*.

### Heme Increases *L. infantum* Infection and Inflammatory Activation of Human Neutrophils

**Figure 3:**
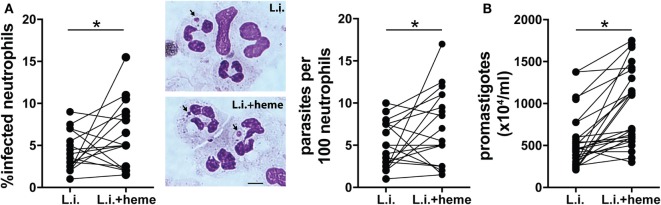
Heme increases viability of *L. infantum* in human neutrophils. Human neutrophils were infected with *L. infantum* (1:5) in the absence or presence of heme (30 µM) for 3 h. The parasite load was measured by optical microscopy at 3 h postinfection as described in the Section “Materials and Methods.” **(A)** The percentage of infected neutrophils and the number of amastigotes per 100 neutrophils are displayed. Representative images of amastigotes inside neutrophils using Diff-Quick staining are shown on the right. Black scale bars, 10 µm. **(B)** Culture supernatants were replaced by Schneider’s medium and viable promastigotes were counted after 24 h. The numbers of viable promastigotes (×10^4^/ml) are shown. Each point on the graphs represents a donor. Data was compared using the Mann–Whitney *U* test (***p < 0.01).

**Figure 4:**
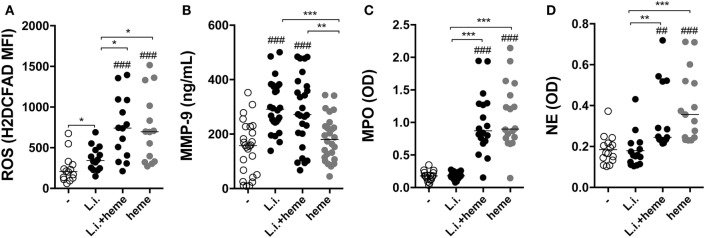
Heme promotes inflammatory activation of human neutrophils. Reactive oxygen species (ROS) production was assessed in human neutrophils by flow cytometry using the probe H2DCFDA **(A)**. Culture supernatants were used to assess production of metalloproteinase 9 (MMP-9) **(B)**, as well as induction of myeloperoxidase (MPO) **(C)** and neutrophil elastase (NE) **(D)** using an enzymatic activity assay. Each point on the graphs represents a donor, and the bars represent median values. Asterisks indicate significant differences tested using the Kruskal–Wallis test with Dunn’s post-test. *p < 0.1, **p < 0.01, ***p < 0.001. Statistical comparison between control groups and experimental groups are shown as #.

Further experiments evaluated how heme could interfere in the viability of *L*. *infantum* inside neutrophils. We found a dose-dependent increase in intracellular parasite burden in neutrophils (Figure S1 in Supplementary Material). Incubation of infected neutrophil cultures with 30 µM heme resulted in increases in both the percentage of infected neutrophils and the number of intracellular parasites (Figure 3A). In addition, substantial increase in the viability (Figure 3B) of *L. infantum* inside neutrophils was detected in cultures treated with heme compared with those observed in untreated cultures. We next analyzed the activation status of infected neutrophils by means of ROS production and release of enzymes from neutrophilic granules. Heme is described as a strong inducer of ROS production in several models, especially in human neutrophils (8). Flow cytometry analysis revealed a significant increase in intracellular ROS generation in infected neutrophils as well as in heme-treated infected neutrophils (Figure 4A). The release of neutrophilic enzymes was further assessed in the supernatants of the same cell cultures. *L. infantum* alone triggered substantial release of MMP-9 from neutrophils (Figure 4B) but did not induce activity of MPO and NE (Figures 4C,D). Cultures treated with heme alone exhibited increased MPO and NE enzymatic activity without affecting MMP-9 secretion compared to that detected in untreated cultures. When the infection of neutrophils occurred in the presence of heme, we observed a mixed pattern hallmarked by a simultaneous increase in MMP-9 levels as well as in MPO and NE activity (Figures 4B–D). The results so far indicate that heme drives specific changes in the inflammatory profile of neutrophils.

### Heme Drives Neutrophil Activation during *L. infantum* Infection

**Figure 5:**
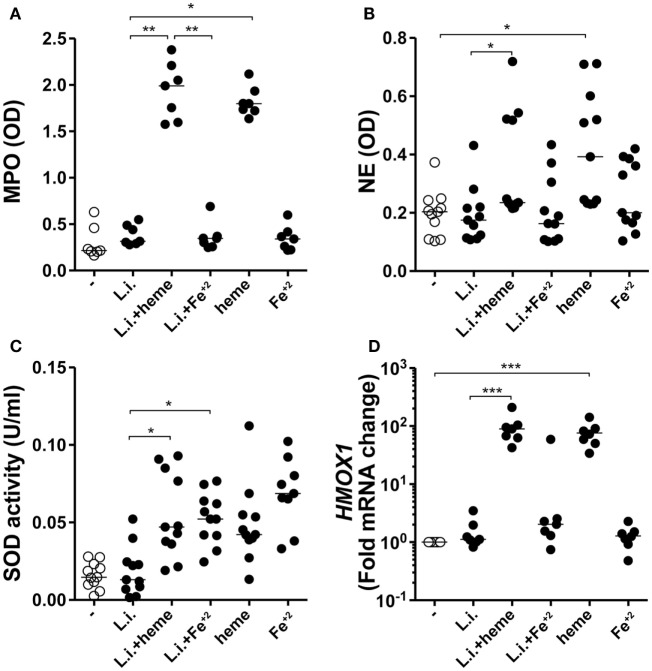
Heme and Fe^+2^ induce neutrophil activation and antioxidant mechanisms in infected neutrophils. Human neutrophils were infected with *L. infantum* in the presence of heme (30 µM) or Fe^+2^ (100 µM). The culture supernatants were used to assess production of myeloperoxidase (MPO) **(A)** and neutrophil elastase (NE) **(B)** using an enzymatic activity assay. Superoxide dismutase (SOD) activity was measured in the culture supernatants as described in Section “Materials and Methods” **(C)**. Heme oxygenase-1 (HO-1) mRNA expression was analyzed by real time-PCR **(D)**. Each point on the graph represents a donor, and the bars represent the median vales. Data were compared using the Kruskal–Wallis test with Dunn’s post-test. *p < 0.1, **p < 0.01, ***p < 0.001.

Infected neutrophil cultures treated with heme displayed substantial increase in both MPO (Figure 5A) and NE (Figure 5B) activity whereas treatment with iron failed to induce such effects. It has been shown that the induction of superoxide dismutase 1 (SOD-1) favors *Leishmania* persistence (29), and that SOD-1 inhibition leads to enhanced parasite killing by human macrophages (30). In the present study, we observed that incubation of the infected neutrophil cultures with either heme or Fe^+2^ resulted in additional increases in SOD-1 activity compared with untreated infected cultures (Figure 5C). In previous studies, the effect of heme in promoting *Leishmania* growth has been linked to the direct induction of HO-1, as an anti-inflammatory mechanism (12, 31). We tested if the observed findings were linked to HO-1 expression in infected neutrophils. After 3 h of infection, *Hmox1* mRNA expression levels were not significantly induced in infected neutrophils treated or not with Fe^2+^ (Figure 5D). As expected, the presence of heme significantly increased transcription for *Hmox1* in infected human neutrophils (Figure 5D). These findings suggest that iron is likely dispensable for the release of granule enzymes, as well as for induction of HO-1.

### The Effects of Heme on *L. infantum* Infection burden in neutrophils Are dependent on Iron and oxidative stress

**Figure 6:**
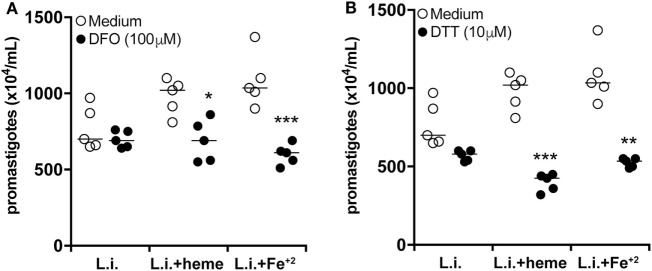
Heme affects neutrophil activation profile during *L. infantum* infection independent of iron. Human neutrophils were infected with *L. infantum* (1:5) in the absence or presence of heme (30 µM) or free iron (Fe^+2^, 100 µM) and co-treated with the iron chelator deferoxamine (DFO) **(A)** or the reactive oxygen species (ROS) scavenger dithiothreitol (DTT) **(B)** for 3 h. The supernatant was then replaced by Schneider’s medium and after 24 h viable promastigotes were counted as described in Section “Materials and Methods.” The numbers of viable promastigotes (×10^4^/ml) are plotted. Each point on the graph represents a donor, and the bars represent the median vales. Data were compared using the Kruskal–Wallis test with Dunn’s post-test. *p < 0.1, **p < 0.01, ***p < 0.001.

Iron has been described to promote growth of bacteria, parasites, and other microorganisms *in vitro* (32). We then tested if iron could be the key driver of the increased parasite burden in infected neutrophils in our *in vitro* system. We observed that treatment of infected cultures with either heme or Fe^+2^ resulted in increased parasite survival compared to untreated infected cultures (Figure 6A). Furthermore, treatment of neutrophil cultures (stimulated with either heme or Fe^+2^) with an iron-chelating agent [deferoxamine (DFO)] resulted in marked decrease in the intracellular parasite viability (Figure 6A). These results confirm the hypothesis that iron is an important factor driving heightened parasite burden in infected neutrophils. Accumulation of intracellular iron is usually linked to oxidative stress because Fe^2+^ can amplify ROS generation through Fenton’s reaction (33). We therefore examined if the effects of heme and iron on parasite replication could be due to excessive ROS production. Heme or Fe^+2^ were added to the infected neutrophil cultures in the presence of the antioxidant dithiothreitol (DTT). We found that DTT treatment robustly dampened the intracellular parasite load (Figure 6B), reinforcing the idea that iron-driven oxidative stress is associated with increased *L. infantum* infection burden in neutrophils.

## Discussion

The inflammatory response during VL is characterized by increased concentrations of circulating cytokines and inflammatory mediators, as well as by a distinct blood transcriptional profile (34). VL is associated with hematological dissemination of *Leishmania* parasites as well as occurrence of hemolytic disorders (35). The pancytopenia is characteristic of this disease, with significant reduction of red blood cells and neutrophils (neutrophil < 500 mm^3^) being considered a key element associated with VL clinical severity (2). Another common feature of VL is the sequestration of red blood cells and platelets in the spleen, resulting in significant anemia (36). Sequestered red cells in the spleen are phagocytized and destroyed by myeloid cells from the reticuloendothelial system (hemophagocytosis) (37, 38). In this setting, hemoglobin and other heme proteins are degraded, releasing free heme (36). We have previously described that serum concentrations of both total heme (13) and of HO-1 (12) are substantially elevated in patients with VL than in uninfected healthy endemic controls. Besides that, it has been shown that HO-1 induction favors *L. infantum* replication inside dog macrophages (39). In the present study, performed in the same patient population, we show that serum concentrations of total heme exhibit positive correlation with HO-1 and LDH levels while being negatively correlated with peripheral blood neutrophil counts, suggesting that systemic levels of heme are linked to parameters associated with increased VL disease progression. In addition, we also found a negative correlation between LDH levels and peripheral blood neutrophil counts. It is possible that the presence of higher concentrations of heme could drive the persistence of the systemic inflammation characteristic of VL and trigger inflammatory mechanisms, oxidative stress, and/or cell death in human neutrophils (6, 10, 40, 41).

Having demonstrated associations between increased concentrations of heme and lower neutrophil counts in the peripheral blood, we further employed an *in vitro* model to investigate the effects of heme on cell death of human neutrophils upon *L. infantum* infection, even though heme has by itself a clear effect on neutrophils death regardless of *Leishmania* infection. The mechanisms underlying the increased parasite viability in neutrophils activated by heme are not completely understood. It is possible that increased cell death induced by heme may have driven/favored parasite survival. Our results clearly show increase in heme concentrations in VL patients. Our working hypothesis is that there is an increased heme-driven neutrophil cell death *in vivo* in VL patients, which favors *Leishmania* persistence. We detected increased cell membrane damage/cytolysis in cultures treated with heme, as assessed by quantification of LDH release in supernatants. In addition, we carefully evaluated cell death by flow cytometry using both Annexin V (to infer apoptosis) and 7AAD (to infer necrosis) staining. Of Note, Arruda et al. (7) have described that heme inhibits human neutrophil apoptosis, assessed by microscopy as well as Annexin V staining, after 20 h of incubation *in vitro* (7). We believe the discrepancies between findings from our and this latter study may result from employment of different experimental models and readouts. Several investigations from our group and others have reported that human neutrophils isolated by gradient methods undergo spontaneous apoptosis in as early as 6 h (21, 42, 43). Thus, evaluation of neutrophil cell death at later timepoint could compromise cell death readout. In addition, our data clearly show a pro-necrotic effect of heme on neutrophils. Importantly, such results are in agreement with other published data in the literature which demonstrate that heme induces neutrophil extracellular traps (NET) formation in neutrophils (44), necroptosis in macrophages (28) and cell death in other leukocytes (45). Further studies using more detail descriptions and different techniques may be warranted to provide a definite elucidation on the discrepancies reported with regard to the effect of heme on neutrophils *in vitro* and *in vivo*.

In the present study, we have also evaluated the effect of heme on the parasite burden and viability of *L. infantum* inside neutrophils. Culturing infected neutrophils in the presence of heme resulted in increased intracellular parasite survival, while displaying a robust inflammatory profile by means of increased production of ROS and release of MMP-9, as well as augmented MPO and NE activity. It is possible that infection-driven oxidative stress is amplified in the presence of heme, which may cause increased cell death and favor parasite survival. Thus, the results presented here corroborate with the idea that heme promotes neutrophil respiratory burst, which, in the context of *Leishmania* infection results in parasite persistence. This concept that oxidative stress favors viability and replication of the infecting pathogen has been shown in other experimental settings and with other microorganisms (46–48).

Another mechanism potentially driving increased parasite survival inside neutrophils could involve the iron pathway. Heme is an important source of iron in several cellular processes (45). In addition, a diverse range of pathogens, including *Leishmania* species, employs capture of host heme and/or iron as an important survival mechanism (49–51). In the present study, supplementation of cell cultures with Fe^+2^ caused increase of the *Leishmania* viability inside neutrophils, arguing the parasite utilizes Fe^+2^ as nutritional source. Interestingly, iron boosted *Leishmania* survival in neutrophils without affecting the cellular release of enzymes from neutrophilic granules. Thus, these observations indicate that the mechanisms inducing activation of other neutrophil effector molecules may be distinct in this experimental model. Furthermore, although the effects on parasite survival were similar between heme and Fe^+2^, the latter did not induce MPO and NE activity in infected neutrophils. This discrepancy between heme and Fe^+2^ potentially underlies different requirements for pathways linked to neutrophil activation and degranulation and promotion of *Leishmania* survival.

Herein, we found that heme triggers a strong intracellular oxidative response in infected neutrophils, which counterintuitively did not affect parasite survival. Accumulation of ROS, and especially superoxide anions, can react with Fe^+2^ resulting in amplification of free radicals (Fenton’s reaction) (52). In excess, ROS are highly detrimental to both parasites and host cells. To circumvent the oxidative stress, host cells produce antioxidants, such as SOD-1, which catalyzes the dismutation of superoxide into oxygen and hydrogen peroxide (53). It has already been demonstrated that the production of SOD-1 by the host cell may provide protection against intracellular pathogens. Interestingly, some parasites can induce antioxidant responses as an escape mechanism. In human macrophages, *L. amazonensis* infection is followed by dramatic increases in SOD-1 expression and activity, which has been shown to directly favor parasite survival (29). In our experiments, *L. infantum* infection itself did not trigger increased SOD-1 activity in human neutrophils. Nevertheless, we observed a remarkable induction of SOD-1 in infected neutrophils treated with either heme or Fe^+2^. The fact that we have also detected accumulation of intracellular ROS in the same experimental conditions associated with SOD-1 induction argues that the antioxidant response was probably insufficient to completely revert the oxidative stress, resulting in further cell death.

Another important antioxidant which has been implicated in the pathogenesis of leishmaniasis is HO-1. We have recently shown that HO-1 induction in macrophages upon *L. infantum* infection is a critical mechanism favoring the persistence of the parasites (12). Whether HO-1 influences *Leishmania* infection in neutrophils is currently unknown. Our results from the clinical study clearly indicate that the serum concentrations of this antioxidant enzyme directly correlated with the levels of total heme. The *in vitro* experiments performed in infected neutrophils demonstrated that HO-1 mRNA was significantly induced only in the presence of heme, highlighting differences in the capacity to produce HO-1 between macrophages and neutrophils. Notably, in our patient cohort, serum levels of heme were also inversely correlated with blood neutrophil counts. We speculate that heme overload in the presence of *L. infantum* infection drives neutrophil hyperactivation and cell death, which in turn, favors parasite survival *in vivo*. These events may be directly linked to disease outcomes, but additional studies are warranted to investigate the interplay between parasite survival, heme-driven inflammation and cell death in severe VL.

## Materials and Methods

### Ethics Statement

This study was carried out in accordance with the recommendations of Institutional Review Board of the Federal University of Sergipe, Brazil with written informed consent from all subjects. All subjects gave written informed consent in accordance with the Declaration of Helsinki. The protocol was approved by the Institutional Review Board of the Federal University of Sergipe, Brazil (license number: 04587312.2.0000.0058).

### Reagents

The heme used *in vitro* assays was commercially obtained from Frontier Scientific (Logan, UT, USA). Drug was diluted immediately before use in 0.1 N sodium hydroxide (NaOH) and RPMI 1640 medium (Gibco, Carsbad, CA, USA) and adjusted to the concentration of 30 µM. DTT, DFO, Iron (II) sulfate hydrate (FeSO4.7H2O) and hemin for parasite culture were commercially obtained from Sigma-Aldrich (New Road, Gillingham, United Kingdom). FeSO4.7H2O was diluted in distilled water and adjusted to a concentration of 100 µM, the compound in solution releases the ferrous iron ions (Fe^+2^). TritonX-100 was purchased from Amersham Biosciences (Piscataway, NJ, USA). Cytotoxicity Detection Kit was from Roche Applied Science (Penzberg, Germany). LDH ELISA kit was from Wuxi Douglin Sci. (Wuxi, China). H2DCFDA fluorescent probe was from Invitrogen/Molecular Probes (Grand Island, NY, USA). Superoxide dismutase-1 (SOD-1) activity kit was from Cayman Chemical Company (Ann Harbor, MI, USA). Aminoactinomycin D (7-AAD) and Annexin V-labeled Ab were purchased from BD Biosciences (San Jose, CA, USA).

### Quantification of Serum LDH, Total Heme, and HO-1 as well as Neutrophil Counts in VL Patients

Serum of patients with VL (*n* = 33) and healthy controls (*n* = 25) was obtained from an endemic area in northeastern Brazil. The clinical and epidemiological characteristics of the study population have been previously described in detail (12); a subset of patients, from whom serum samples as well as information on neutrophil counts were available, was used in the present study. The quantification of serum levels of LDH, total heme and HO-1, as well as peripheral neutrophil counts were performed before antileishmanial therapy. Data on neutrophil counts were obtained from WBC counts in a clinical laboratory from the endemic area. HO-1 was measured using the ELISA kit from Enzo Life Sciences (Farmingdale, NY, USA). LDH was measured in serum samples using the ELISA kit from Wuxi Douglin Sci. (Wuxi, China); and in supernatant samples using Cytotoxicity Detection Kit from Roche Applied Science (Penzberg, Germany). Total heme was estimated by a colorimetric determination, using the QuantiChrom Heme Assay Kit (BioAssay Systems, Hayward, CA, USA) following the manufacturer’s instructions.

### Parasite Culture

*Leishmania infantum* promastigotes (MCAN/BR/89/Ba262) were grown in hemoflagellate-modified minimal essential medium supplemented with 10% (v/v) fetal bovine serum (FBS) and 24.5 mM hemin at 23°C (BOD incubator). In all experiments, the cultures of *L. infantum* were used at stationary phase.

### Infection Assays

Human blood was obtained from volunteers at the Hemocentro do Estado da Bahia, BA, Brazil. Human neutrophils were isolated by gradient separation with polymorphonuclear medium (PMN) according to manufacturer’s instructions (Axis-ShieldPoc AS, Oslo, Norway). Neutrophils were collected and washed three times with saline at 4°C for 10 min at 1300 RPM. Neutrophils were plated with RPMI-1640 medium, supplemented with 1% Nutridoma-SP, 2 mM L-glutamine, 100 U/ml penicillin, and 100 µg/ml streptomycin. Cells were infected with stationary *L. infantum* promastigotes (five parasites for one neutrophil) in the presence or absence of indicates concentrations of heme. In some experiments, neutrophils were also incubated with 10 µM DTT or 100 µM DFO. The cultures were incubated for 3 h at 37°C, 5% CO_2_ and infection burden was evaluated subsequently upon infection. Subsequent to infection in the presence or absence of heme or Fe^+2^, intracellular load of *L. infantum* in neutrophils was evaluate through viability of the parasites using the Schneider method adapted from Ribeiro-Gomes and collaborators (54). Briefly, after 3 h of incubation the infected neutrophils were centrifuged at 1,300 rpm for 10 min at 4°C and the supernatant containing not internalized promastigotes was discarded and replaced by 250 µl of Schneider medium supplemented with 20% FBS and 1% l-glutamine (2 mM), penicillin (100 U/ml), and streptomycin (100 µg/ml). Then, the infected neutrophils were incubated at 23°C and after 24 h of incubation proliferating extracellular viable promastigotes were counted using Neubauer chamber. Alternatively, intracellular load of *L. infantum* was estimated using cytospin preparations stained with Diff-Quick. Number of infected neutrophils and intracellular amastigotes was quantified under light microscopy in 200 neutrophils per slide.

### Evaluation of Neutrophil Activity

After 3 h of incubation, the supernatants of infected neutrophil cultures in presence or absence of heme or Fe^+2^ were used to test the production of metalloproteinase-9 (MMP-9) by sandwich ELISA according to the manufacturer’s instructions (R&D systems, Minneapolis, MN, USA). The enzymatic activity of the MPO and NE was evaluated under the same experimental conditions using a specific substrate for each enzyme, according to a previously described protocol of Ref. (55). The results were assessed using spectrophotometry by detecting the optical density of each well at 410 nm for MPO and at 492 nm for NE, respectively.

### Assessment of Oxidative Response

Superoxide dismutase-1 activity was detected in cell free supernatant, according to the manufacturer’s instructions (Cayman Chemical Company, MI, USA). In order to evaluate the presence of superoxide, neutrophils infected in the presence of heme or Fe^+2^ were treated with hydroxylamine (500 µM) for 3 h. Hydroxylamine which was oxidized to nitrite was measured by spectrophotometry in the absorbance 560 nm using the optical density. For detection of ROS, subsequent to 1 h of infection in the presence of heme or Fe^+2^, cells were stained with 10 µM H2DCFDA fluorescent probe following analyses by FACS (Fortessa LSR II, BD Biosciences, Franklin Lakes, NJ, USA), according to the manufacturer’s instructions. The geometric median fluorescence intensity was evaluated using FlowJo software (Tree Star, Ashland, OR, USA).

### HO-1 mRNA Expression

Extraction of ribonucleic acid (RNA) in neutrophils infected with *L. infantum* (3 h post-infection) in presence or absence of heme was performed using Trizol Reagent (Invitrogen, Life Technologies, Carlsbad, CA, USA) according to the manufacturer’s instructions. Reverse transcription was performed with 250 ng of total RNA using the High Capacity cDNA Reverse Transcription Kit (Applied Biosystems™, Foster City, CA, USA). The obtained cDNA was stored at −20°C until further use. The qPCR was performed in an ABI 7500 FAST Real-Time PCR equipment (Applied Biosystems™, Foster City, CA, USA). The amplification reactions were performed with Power SYBR^®^ Green, according to the manufacturer’s instructions. The standard qPCR conditions were as follows: 10 min at 95°C, followed 40 cycles at 95°C for 15 s, 60°C for 60 s. The primers used for qPCR reactions were HO-1 (Fw: AGG CCA AGA CTG CGT TCC T and Rev: GGT GTC ATG GGT CAG CAG C) and for endogenous controls GAPDH (Fw: CAC ATG GCC TCC AAG GAG TAA and Rev: TGA GGG TCT CTC TCT TCC TCT TGT) and β-actin (Fw: CCT GGC ACC CAG CAC AAT and Rev: GCC GAT CCA CAC GGA GTA CT). After amplification and dissociation curve runs, the values of threshold cycle (Ct) were obtained with the aid of the Operational Programme 7500™ System (Applied Biosystems, USA). The intra-assay precision was calculated using the equation E (−1/slope) to confirm precision and reproducibility of qPCR. The expression levels were normalized based on the geometric mean of endogenous controls. Relative expression folds were calculated based on 2^ddCt^ method (56).

### Neutrophil Viability Assays

Neutrophil viability was quantified by the expression of membrane phosphatidylserine detected by annexin V binding using annexin V-FITC and 7-aminoactinomycin D nucleic dye as recommended by the manufacturer (BD Pharmingen). For LDH release assay, neutrophils (5 × 10^4^ cells/well) were cultured with 30 µM of heme, and infected with *L. infantum* promastigotes. The release of LDH was measured 3 h post heme treatment in cell-free culture supernatant by the Cytotoxicity Detection Kit. Blank LDH levels were subtracted from experimental LDH values and total LDH activity was determined by lysing the cells with 1% Triton X. Results are displayed as percent of total LDH release.

### Statistical Analysis

Statistical analyzes were performed using GraphPad Prism program 5.0 (GraphPad Software, San Diego, CA, USA). Comparisons were tested using the nonparametric Kruskal–Wallis test (for more than two experimental groups) with Dunn’s multiple comparisons post-test or linear trend analysis. The Mann–Whitney *U* test was used to compare median values between two groups. Correlations were tested using the Spearman rank test. Frequencies were compared using the Fisher’s exact test. Differences were considered statistically significant when *p* < 0.05. All the experiments were performed at least two times, using on average 5–6 volunteers per experiment (cells from each one of the 5–6 volunteers were exposed to different experimental conditions in each experiment).

## Ethics Statement

This study was carried out in accordance with the recommendations of Institutional Review Board of the Federal University of Sergipe, Brazil with written informed consent from all subjects. All subjects gave written informed consent in accordance with the Declaration of Helsinki. The protocol was approved by the Institutional Review Board of the Federal University of Sergipe, Brazil (license number: 04587312.2.0000.0058).

## Author Contributions

GQ-C, NL, MB, BA, and VB conceived and designed the study. GQ-C, NL, FC, DZ, DA, DM, NT, and DP, performed the experiments. GQ-C, NL, CB, DP, MG, CO, RA, MB, BA, and VB contributed with data analysis. DM, RA, MB, BA, and VB provided materials and infrastructural support. GQ-C, NL, MB, BA, and VB wrote and revised the manuscript.

## Conflict of Interest Statement

The authors declare that they do not have a commercial association that might pose a conflict of interest.
