# Supplementary material for: Heme Drives Oxidative Stress-Associated Cell Death in Human Neutrophils Infected with Leishmania infantum
Source: Front Immunol. 2017 Nov 23;8:1620. doi: 10.3389/fimmu.2017.01620 (PMC5703736; doi:10.3389/fimmu.2017.01620)
Supplement: Supplementary file 1 [file Image_1.PDF]

Supplementary Material

Heme Drives Oxidative Stress-associated Cell Death in Human Neutrophils Infected with *Leishmania infantum*

Graziele Quintela-Carvalho; Nívea F. Luz; Fabiana S. Celes; Dalila L. Zanette; Daniela Andrade; Diego Menezes; Natália M. Tavares; Claudia I. Brodskyn; Deboraci B. Prates, Marilda S. Gonçalves; Camila I. de Oliveira; Roque P. Almeida; Marcelo T. Bozza; Bruno B. Andrade; Valeria M. Borges

Corresponding authors:

Valeria M. Borges

vborges@bahia.fiocruz.br (VMB)

Bruno Bezerril Andrade

bruno.andrade@bahia.fiocruz.br (BBA)

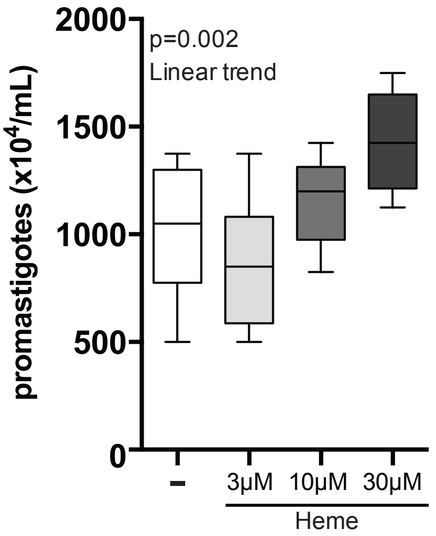

Supplementary Figure 1. Increasing doses of heme favor *Leishmania infantum* replication. Human neutrophils were infected with *L. infantum* (1:5) in the absence or presence of different doses of heme (3, 10 and 30μM) for 3 hours. Supernatants were then replaced by Schneider's medium and viable promastigotes were counted after 24 hours as described in Methods. The numbers of viable promastigotes ($x10^4$/mL) are plotted. Boxes represent medians and interquartile ranges, and whiskers represent maximum and minimum values. Data were compared using the Kruskal-Wallis test with linear trend post-test.
